# Gene Flow and Maintenance of Genetic Diversity in Invasive Mosquitofish (*Gambusia holbrooki*)

**DOI:** 10.1371/journal.pone.0082501

**Published:** 2013-12-16

**Authors:** David Díez-del-Molino, Gerard Carmona-Catot, Rosa-Maria Araguas, Oriol Vidal, Nuria Sanz, Emili García-Berthou, Jose-Luis García-Marín

**Affiliations:** 1 Laboratori d’Ictiologia Genètica, Departament de Biologia, Universitat de Girona, Girona, Spain; 2 Institut d'Ecologia Aquàtica, Departament de Biologia, Universitat de Girona, Girona, Spain; Instituto de Higiene e Medicina Tropical, Portugal

## Abstract

Genetic analyses contribute to studies of biological invasions by mapping the origin and dispersal patterns of invasive species occupying new territories. Using microsatellite loci, we assessed the genetic diversity and spatial population structure of mosquitofish (*Gambusia holbrooki*) that had invaded Spanish watersheds, along with the American locations close to the suspected potential source populations. Mosquitofish populations from the Spanish streams that were studied had similar levels of genetic diversity to the American samples; therefore, these populations did not appear to have undergone substantial losses of genetic diversity during the invasion process. Population structure analyses indicated that the Spanish populations fell into four main clusters, which were primarily associated with hydrography. Dispersal patterns indicated that local populations were highly connected upstream and downstream through active dispersal, with an average of 21.5% fish from other locations in each population. After initially introducing fish to one location in a given basin, such dispersal potential might contribute to the spread and colonization of suitable habitats throughout the entire river basin. The two-dimension isolation-by-distance pattern here obtained, indicated that the human-mediated translocation of mosquitofish among the three study basins is a regular occurrence. Overall, both phenomena, high natural dispersal and human translocation, favor gene flow among river basins and the retention of high genetic diversity, which might help retain the invasive potential of mosquitofish populations.

## Introduction

Biological invasions are a central component of global change, and a major threat to the biodiversity of freshwater ecosystems [1]. Invaders may change radically the functioning of an ecosystem, to which they are introduced, resulting in the decline or extinction of native species through predation, competition, and habitat alteration [2]. Often, biological invasions begin when humans introduce a few individuals of a species to a new environment. Once established, the new population spreads to neighboring locations by natural dispersal. For example, the Eurasian spread of the topmouth gudgeon (*Pseudorasbora parva*) began accidentally when humans introduced it during the establishment of new cultured stocks of the common carp (*Cyprinus carpio*), and was followed by further natural dispersal of short distances [3]. Genetic variation is closely linked to the success of biological invasions [4]. When introductions begin with just a few individuals, reduced genetic diversity is expected during the first stages of the invasion [5], [6]. However, several studies have shown high diversity in populations at later stages of the invasion process (see [7]). The recovery of genetic diversity in invaded territories might result from gene flow between recently established populations within the invaded range that have become increasingly interconnected. Multiple introductions from divergent stocks also contribute towards increasing local diversity in invaded territories [8], particularly if these introductions occur separately in time from the initial founder event (e.g., [9]).

The eastern mosquitofish, *Gambusia holbrooki,* is one of the most commonly introduced freshwater species [10]. At present, established populations of this species in Europe, Africa, Asia, and Australia are causing local extinction and decline of several native fish and amphibian species [11], [12]. For example, the aggressive behavior of *G. holbrooki* has caused a decline in the feeding rates and reproductive success of two Iberian endemic fish species, *Valencia hispanica* and *Aphanius iberus* [13]. Similarly, Carmona-Catot et al. [14] showed that introduced *G. holbrooki* were able to competitively displace *A. iberus* populations. Mosquitofish introductions were originally supported by governmental health agencies to control mosquito populations, which are vectors of various diseases, such as malaria [15]. In Europe, 12 individuals of *G. holbrooki* were initially introduced into a pond in southern Spain in 1921 [16]. Subsequently, humans spread *G. holbrooki* throughout the Mediterranean basin [17]. Despite their small size, mosquitofish are extremely successful in new environments [15]. Both the invertivorous diet and wide ecological tolerance of *G. holbrooki* have probably contributed to its successful integration into Iberian fish communities [18].

Temporal fluctuations in population size reduce the average effective population size (*Ne*), with reduced effective sizes intensifying the loss of diversity due to genetic drift [19]. Although there is a major decline in the size of mosquitofish populations during winter after the summer flush [20], several studies have indicated high genetic diversity within American populations that often exceeds average values described for freshwater fishes [21]. The high reproductive potential generated by overwintering pregnant females, multiple paternity, and offspring reaching maturity within a few weeks probably contribute towards maintaining large effective population sizes and preventing the loss of population diversity [22]–[24]. Moreover, gene flow between seasonally isolated demes favors population diversity in large territories [25]. For instance, sporadic individual exchange among close populations prevents divergence among collections within basins in invaded territories [26].

Several models, such as isolated populations with no current migration and metapopulations of ephemeral populations connected by gene flow, may explain the population structure of organisms in linear river systems (e.g., [27]). Native mosquitofish populations usually represent single breeding units [28], while large transects within a river basin are occupied by a single population with ephemeral local subpopulations [29]. Source-sink dynamics are sometimes responsible for the population structure of mosquitofish (e.g., [25], [30]). Along a river system, dominant downstream gene flow increases the genetic diversity of lowland populations [31], [32].

DNA molecular markers contribute to improving our understanding of evolutionary changes that occur during biological invasions [33]–[36]. Highly polymorphic microsatellite loci provide the discrimination required to address questions about population structure and gene flow [24], [27]. In this study, we used microsatellite loci to evaluate putative losses of genetic variation during introductions of *G. holbrooki*. We aimed to understand the mechanisms that contribute towards retaining levels of diversity within populations inhabiting invaded rivers compared to populations in native basins.

## Materials and Methods

### Ethics statement

Animal samples were collected and manipulated under a permit (SF/012/2011) provided by the Agriculture, Fisheries, Food and Environment Department of the Autonomous Community of Catalonia. All work was performed in compliance with and approved by the Ethics Committee of the University of Girona and meets the requirements stated by the Spanish (RD53/2013) and Catalonian (D214/1997) laws of animal care, and experimentation.

### Sample collection

**Figure 1:**
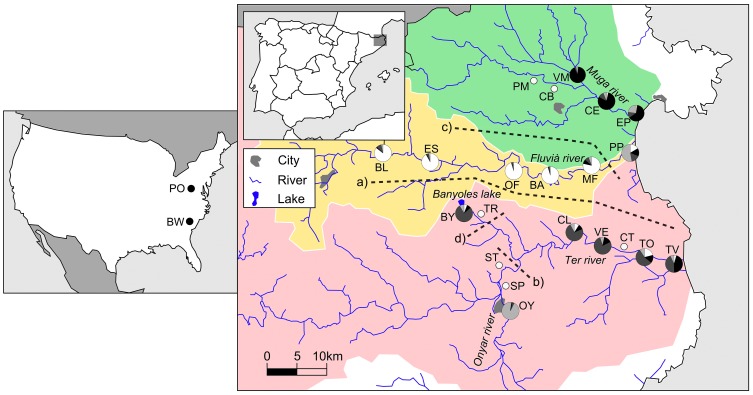
Geographical location of the collection sites. Sampled sites where *G. holbrooki* was not found are indicated with empty circles. Grey-scaled pie charts (white, light grey, dark grey, and black) represent mean proportional ancestry of every sampled site attributed to each cluster inferred by STRUCTURE. Watersheds are colored. Dotted lines represent geographical barriers indicated by BARRIER and the letters indicate the order in which the program detected these barriers. Location codes are presented in Table 1. (A detailed map with information about the road network is available at: http://mapsengine.google.com/map/viewer?mid=zgd4mwb-ESLE.kSE9TUfF2uQ4).

A total of 556 *G. holbrooki* were collected from 15 sites along three watersheds (Muga, Fluvià, and Ter rivers) in northeastern Spain. The largest of these rivers is the Ter, with a basin area of 2955 km^2^, with its headwaters in the Pyrenees and its upper course being partially snow-fed. The Fluvià (974 km^2^) and Muga (758 km^2^) are typical Mediterranean streams with smaller watersheds, and have their headwaters located in mountainous areas. All three rivers are subject to a Mediterranean climate, with severe summer droughts and autumn floods [37]. The Ter and Muga rivers have many small weirs, along with a few large dams that form major barriers, altering connectivity among fish populations, whereas the Fluvià only has weirs. Mosquitofish are currently absent from the upper course of these watersheds; hence, we collected samples from the middle and lower courses of these three watersheds (Fig. 1, Table 1). Sampling sites were shallow areas (<1.5 m depth) along the riverbank, with low water velocity and dense vegetation, usually reed beds (*Phragmites australis*). We also analyzed 36 individuals from the Potomac River (Washington) and 16 individuals from Brunswick (North Carolina), because it has been suggested that these populations are the closest to the main American source of *G. holbrooki* individuals that were introduced to Europe [16].

**Table 1:** Description of the study locations.

Basin	Location	Code	Coordinates	Date	N
Muga	Pont de Molins	PM	2°57'11.49'', 42°18'9.41''	24/08/2010	ND
	Cabanes	CB	2°58'40.08", 42°17'55.68"	24/08/2010	ND
	Vilanova de la Muga	VM	3°2'29.38'', 42°16'49.86''	24/08/2010	40 (20)
	Castellò d'Empúries	CE	3°4'16.16'', 42°15'17.54''	24/08/2010	40 (20)
	Empuriabrava	EP	3°7'26.78'', 42°14'14.97''	24/08/2010	40 (20)
Fluvià	Besalú	BL	2°44'9.012'', 42°11'27.41''	24/08/2010	40 (27)
	Esponellà	ES	2°47'41.24'', 42°11'0.268''	24/08/2010	40 (20)
	Orfes	OF	2°52'12.54'', 42°10'14.20''	24/08/2010	40 (20)
	Báscara	BA	2°54'51.88'', 42°9'49.76''	24/08/2010	40 (20)
	Sant Miquel de Fluvià	MF	3°0'46.72'', 42°9'56.04''	24/08/2010	40 (20)
	Sant Pere Pescador	PP	3°4'18.02'', 42°10'44.81''	24/08/2010	27 (12)
Ter	Banyoles	BY	2°44'54.49'', 42°7'7.317''	29/07/2010	40 (20)
	Terri	TR	2°46'39.00', 42°7'1.704''	29/07/2010	ND
	Onyar	OY	2°49'48.00'',41°58'25.53''	29/07/2010	40 (22)
	Sant Ponç	SP	2°49'20.61'',41°59'33.67''	29/07/2010	ND
	Sarrià de Ter	ST	2°49'33.37'',42°0'49.66''	29/07/2010	ND
	Colomers	CL	2°59'8.999'',42°4'58.51''	29/07/2010	40 (20)
	Verges	VE	3°2'38.79'',42°3'11.45''	29/07/2010	11 (6)
	Canet de la Tallada	CT	3°4'5.232'',42°2'29.67''	29/07/2010	ND
	Torroella de Montgrí	TO	3°9'7.177'',42°1'31.77''	29/07/2010	40 (20)
	Ter Vell	TV	3°11'43.51'',42°2'42.84''	29/07/2010	38 (34)
America					
	Potomac River	PO	38°38'60.0'',77°11'0.0''	02/03/2009	36 (25)
	Brunswick	BW	34°16'60.0'',78°29'0.0''	07/11/2007	16 (7)

ND: Gambusia holbrooki not detected.

Geographical coordinates: all longitudes are East, and latitudes North. N: sample size (females).

*Gambusia holbrooki* specimens from Iberian rivers were collected from the riverbank using dip nets. All samples were collected from July to August 2010 and, to minimize any seasonal effects on population demography, only adult individuals born during the spring of the same year were selected by discarding females with a standard length of less than 2.5 cm and more than 3.5 cm, and males with a body length of less than 2.0 cm [38]. Individuals were classified as adult males if a fully formed gonopodium was present, and as females if not. We attempted to collect 20 males and 20 females from each site; however, adult fish availability modified this ratio (Table 1). Whole fish were euthanized by lethal sedation *in situ*, and then preserved in 96% ethanol until DNA was extracted at the laboratory.

### DNA extraction and microsatellite analyses

For each fish that was collected, genomic DNA was isolated from the caudal muscle using the Realpure Genomic DNA extraction toolkit (Durviz SL, Valencia, Spain) following the manufacturer’s instructions. Genomic DNA was stored at –20°C until further use in Polymerase Chain Reactions (PCRs). Variation was analyzed at 11 loci (*Pooc-G49*, *Mf13*, and *Gafμ3*, *Gafμ5*, *Gafμ6*, *Gafμ7, Gaaf7, Gaaf9, Gaaf10, Gaaf13, and Gaaf15*), with two optimized multiplex PCR as described in Diez-del-Molino et al. (submitted). Both multiplex PCR were conducted under the same conditions: 30 µl of reaction volume containing 5–15 ng genomic DNA, 0.34 µM of each primer, 200 µM dNTPs, 1.5 mM MgCl_2_, and 0.75 units of Taq polymerase. The PCR cycling conditions were as follows: initial denaturation at 94°C for 3 min, followed by 35 cycles of 30 s at 94°C, 90 s at 60°C, 90 s at 72°C, and ending with a final extension of 10 min at 72°C. Forward primers were fluorescently labeled, and genotype peaks were resolved on a 3130 Genetic Analyzer and using GeneMapper 4.0 software (Applied Biosystems, Foster City, CA, USA).

### Genetic diversity within locations

Genetic diversity within each study site was estimated from direct counts as the mean observed heterozygosity (*H_O_*) and the number of alleles per locus (*A*). Genetic diversity was also measured using the estimated expected heterozygosity (*H_E_*) and allelic richness (*r*) from allele frequencies using FSTAT 2.9.3 [39]. Using GENEPOP 4.0 [40], we measured the Hardy-Weinberg equilibrium (HWE) at each site, and tested for linkage disequilibrium between all pairs of loci. We corrected for multiple comparisons using the sequential Bonferroni test [41]. The presence of null alleles was detected using MICROCHECKER 2.2.3 [42], and their frequencies were estimated in FREENA [43]. We tested for recent population bottlenecks at the study sites using BOTTLENECK 1.2.02 [44].

### Genetic structure within and among rivers

Pairwise population differentiation (*F_ST_*) and significance values were calculated using FSTAT software. To assess the relevance of stepwise mutations on population differentiation (*R_ST_*), an allele permutation test was performed with 1000 randomizations that simulated the distribution of allele sizes and *R_ST_* values using SPAGEDI version 1.1 [45]. Allele richness and gene diversity patterns (*H_E_* and *F_ST_*) within basins were compared among basins using permutation tests in FSTAT (1000 permutations). Non-parametric Wilcoxon signed-rank tests were used to compare allele richness and gene diversity (*H_E_*) between upstream and downstream collections within each study basin.

Isolation-by-distance (IBD) within and among watersheds was estimated from the correlation between genetic and geographical distance matrices among sampling sites. We used geographical distances, rather than hydrographical distances; because natural dispersal within linear river basins or human-mediated translocation by road could be involved in the connectivity between locations, given their geographical proximity (less than 50 km on average) and anastomosed road network (Fig. 1). The geographical distances between sample sites were estimated using Google Earth. Pairwise genetic differentiation was linearized as *F_ST_*/(1-*F_ST_*) and geographical distance was log-transformed for these analyses [46]. Significance was determined by Mantel tests with 10000 permutations using the IBD Web service 3.15 [47]. Additional information was obtained from the regression analyses of the estimates of the effective number of migrants (*Nm*) between populations pairs (Nm =  (1 – *F_ST_*)/4*F_ST_*) and their geographical distances (both variables log-transformed). Negative relationships indicate IBD, and the slope (b) of the linear regression (Log(*Nm*)  = a + bLog(d), where d equals geographical distance) is –1 for one-dimensional stepping stones models and –0.5 for the two-dimensional models [48].

The minimum number of homogeneous units (*K*) over sampled individuals was estimated using the MCMC method in STRUCTURE 2.3.3 [49]. Runs for each possible *K* (1 to 15) were repeated 10 times. Each run used a burn-in of 40000 iterations, a run length of 100000 iterations, and the model of independent allele frequencies. The most likely value of *K* was selected following Evanno [50]. The group-level Bayesian analysis in BAPS 5.4 [51] grouped populations that frequently exchanged individuals. BAPS analyses were repeated 10 times, with the maximum number of clusters set to 15. While STRUCTURE results tend to be conservative in the number of clusters detected providing ancestral information related with the history of introductions of the species, BAPS performed better in clustering together populations with recent gene flow (e.g., [52]). In addition, genetic differentiation among populations was depicted by two-dimensional plots from the principal components analysis (PCA) of the allele frequencies matrix in GENALEX 6.4.

Major genetic discontinuities in the study area were assessed using Monmonier’s algorithm in BARRIER 2.2 [53], which detects hidden barriers to gene flow among sites according to their geographical coordinates and relative genetic differentiation (*F_ST_*). These analyses were conducted using the *F_ST_* matrices from single-locus information corrected by the presence of null alleles (FREENA software). We identified the main barriers for each locus, and only retained those confirmed by at least six loci.

Analyses of molecular variance (AMOVA) were conducted in ARLEQUIN 3.5 [54]. Two hierarchical models were tested for partitioning the genetic diversity into three levels: within locations, among locations within regions, and among regions. The first AMOVA model assumed a pure hydrographical pattern of population hierarchy (watersheds  =  regions). Another AMOVA grouped locations according to the main clusters identified by STRUCTURE (clusters  =  regions).

### Gene flow

Contemporary migration rates among populations were estimated by using the Bayesian inference as implemented in BAYESASS 3.0 software [55], which is a method that does not assume migration-drift or Hardy-Weinberg equilibrium. A total of 5×10^6^ iterations were performed until the MCMC chains reached stationarity (i.e., constant over time). Migration parameters were estimated by sampling every 1000 iterations after a burn-in of 10^6^ iterations. Delta values were adjusted following the BAYESASS manual recommendations. Five runs using different starting points were performed, and the results with the highest likelihood were retained.

## Results

### Diversity within locations

At the invaded locations, all 11 microsatellite loci were polymorphic, ranging in variability from just two alleles (*Mf13, Gaaf15,* and *Gaaf9* loci) to nine (*Gaaf13* locus). Average allelic richness (*r*) ranged from 2.45 in ES (Fluvià River) to 3.31 in CO (Ter River) (Table 2). The observed heterozygosity (*H_O_*) ranged from 0.336 in OF (Fluvià River) to 0.475 in PP (Fluvià River), and the expected heterozygosity (*H_E_*) ranged from 0.345 in BA (Fluvià River) to 0.500 in CE (Muga River). At the studied American collections, diversity levels averaged 4.45 for allelic richness, 0.465 for *H_O_*, and 0.557 for *H_E_*. After adjusting for differences in sample sizes, FSTAT permutation tests demonstrated lower allele richness (*P* = 0.009) and *H_E_* (*P* = 0.010) in the invaded Spanish locations compared to the potential American sources. Non-significant *F_ST_* differentiation was detected between males and females (Table 3). Subsequent analyses were then performed pooling both sexes as a single collection for each location.

**Table 2:** Genetic diversity of *Gambusia holbrooki* in the study locations.

Basin	Location code	*A*	*r*	*H_O_*	*H_E_*	*F_IS_*
Muga	VM	3.27	2.68	0.393	0.434	0.094*
	CE	3.63	3.16	0.414	0.500	0.173*
	EP	3.09	2.85	0.442	0.478	0.075*
Fluvià	BL	3.00	2.56	0.389	0.417	0.066
	ES	2.91	2.45	0.371	0.387	0.041*
	OF	3.18	2.53	0.336	0.358	0.061
	BA	3.09	2.51	0.350	0.345	–0.015
	MF	3.55	2.84	0.402	0.420	0.042*
	PP	3.73	3.14	0.475	0.489	0.031
Ter	BY	3.27	2.65	0.434	0.453	0.042
	OY	3.36	2.87	0.449	0.464	0.030
	CL	4.00	3.31	0.438	0.482	0.092*
	VE	2.91	2.85	0.413	0.411	–0.004
	TO	3.55	3.08	0.457	0.474	0.036*
	TV	3.82	3.03	0.404	0.460	0.121
America	PO	5.45	4.80	0.457	0.577	0.213*
	BW	4.27	4.27	0.472	0.537	0.149

*P*<0.05). Significant Hardy-Weinberg disequilibria after Bonferroni correction (

*A*), allele richness (*r*), average observed heterozygosis (*H_O_*), average expected heterozygosis (*H_E_*), and fixation index (*F_IS_*). Location codes are presented in Table 1. Average number of alleles (

**Table 3:** Genetic (*F_ST_* corrected by the presence of null alleles, below the diagonal) and geographical distances (km, above the diagonal) between samples.

		Muga			Fluvià						Ter					
Basin	Code	VM	CE	EP	BL	ES	OF	BA	MF	PP	OY	BY	CL	VE	TO	TV
Muga	VM	–**0.011**	3.67	7.14	29.01	22.87	18.06	16.90	12.78	11.79	29.05	38.40	22.45	24.65	27.94	30.06
	CE	**0.020**	–**0.006**	3.85	31.11	23.96	18.59	17.22	11.65	7.94	29.62	37.24	20.43	22.31	25.01	26.84
	EP	0.060	0.056	–**0.001**	33.85	26.71	21.12	19.01	12.72	6.50	32.16	37.88	20.60	21.61	23.10	24.65
Fluvià	BL	0.303	0.268	0.240	**0.028**	8.28	14.01	17.50	24.07	31.45	8.98	28.26	26.39	32.13	38.97	43.00
	ES	0.293	0.251	0.225	0.037	**0.026**	5.93	9.18	16.07	23.58	6.72	23.83	18.55	24.32	30.97	35.03
	OF	0.320	0.276	0.283	0.094	0.052	**0.010**	3.50	9.96	17.52	11.02	23.29	13.58	19.34	25.68	29.55
	BA	0.313	0.273	0.262	0.122	0.044	**0.021**	–**0.002**	6.93	14.83	13.43	22.34	10.69	16.08	22.51	26.40
	MF	0.221	0.178	0.166	0.131	0.060	0.056	0.043	**0.028**	7.85	20.17	25.57	9.49	13.33	18.21	21.53
	PP	0.146	0.114	0.046	0.195	0.177	0.239	0.220	0.129	–**0.007**	28.07	31.29	14.17	15.02	17.06	19.32
Ter	OY	0.233	0.196	0.124	0.271	0.254	0.337	0.320	0.240	0.090	**0.006**	19.13	17.60	19.32	24.42	27.57
	BY	0.166	0.130	0.125	0.140	0.151	0.184	0.200	0.149	0.126	0.197	–**0.001**	19.36	25.05	31.75	39.96
	CL	0.151	0.107	0.101	0.181	0.146	0.172	0.168	0.097	0.094	0.175	0.061	**0.011**	5.90	12.66	16.43
	VE	0.187	0.142	0.140	0.226	0.194	0.242	0.231	0.146	0.120	0.210	0.108	0.041	–**0.046**	7.00	10.99
	TO	0.135	0.108	0.084	0.153	0.133	0.166	0.158	0.093	0.091	0.176	0.047	0.032	0.064	**0.003**	4.18
	TV	0.108	0.080	0.064	0.244	0.221	0.245	0.234	0.143	0.124	0.204	0.108	0.057	0.105	0.065	**0.066**

*F_ST_* values (*P*>0.05). In bold: non-significant

*F_ST_* divergence between sexes within location. Location codes are presented in Table 1. Diagonal:

Deviations from HWE were detected at seven Spanish locations after Bonferroni correction (Table 2). According to MICROCHECKER, null alleles were responsible for the observed positive *F_IS_* values. Significant null allele frequency was estimated at *Gafμ5* (*q* = 0.071 in ES, Fluvià River; *q* = 0.089 in TO, Ter River), *Gafμ6* (*q* = 0.149 in ES and *q* = 0.231 in CE, Muga River), *Gafμ7* (*q* = 0.172 in MF, Fluvià River), *Gaaf10* (*q* = 0.081 in CE, Muga River; *q* = 0.147 in CO, *q* = 0.126 in TO and *q* = 0.132 in TV, Ter River), and *Gaaf15* (*q* = 0.231 in VM and *q* = 0.265 in CE, Muga River). No significant pairwise linkage disequilibria were observed. According to the BOTTLENECK analyses, heterozygosity excess relative to mutation-drift equilibrium was observed at CE (Muga River), BL, and MF (Fluvià River), and VE, and OY (Ter River); however, the allele-shift model test reported additional signals for a bottleneck at VE only.

### Population divergence within and among basins

No significant differences between *F_ST_* and *R_ST_* estimates were observed (*P* = 0.345), indicating that local mutations have limited effects on population structure. Significant genetic differentiation was detected between almost all population pairs (Table 3), except for two neighboring sites in the Muga (VM and CE locations) and Fluvià (OF and BA locations) rivers (Table 3). Estimated average genetic differentiation among invaded Spanish locations was *F_ST_* = 0.1641, with no substantial change in this estimate after correcting for null alleles (*F_ST_* = 0.1642). The collection OY from the Onyar tributary in the Ter basin had the largest average pairwise *F_ST_* (0.194).

Despite the different values on estimates of average allele richness, the average expected heterozygosity, and population differentiation observed in the Iberian rivers (Table 4), FSTAT permutation tests only indicated marginally reduced heterozygosity in the Fluvià basin compared to the Muga (*P* = 0.062) and Ter basins (*P* = 0.052). Overall, among-basin differences in genetic diversity (allele richness and heterozygosity) were non-significant between upstream and downstream locations. Marginal (*P* = 0.067) increased divergence among the upstream locations of the three basins was indicated (Table 4). Within-basin comparisons with Wilcoxon signed-rank tests indicated higher allele richness for just the downstream location of the Fluvià River (*P* = 0.042). Non-significant differences were obtained in the Muga River basin, and an unexpected higher richness was indicated at the upstream CL location compared to the downstream TV in the Ter River basin (*P* = 0.034). None of these changes in allele richness between upstream and downstream locations resulted in significant differences on the estimated amount of heterozygosity.

**Table 4:** Genetic diversity patterns within and among the studied locations.

Region/basin	*r*	*H_E_*	*F_ST_*
American sources	3.87	0.552	0.242
Spain (all study locations)	2.83	0.432	0.164
Muga River	2.89	0.470	0.042
Fluvià River	2.66	0.397	0.104
Ter River	2.97	0.463	0.116
Upstream (VM, BL, CL locations)	2.85	0.443	0.215
Downstream (EP, PP, TV locations)	3.00	0.473	0.073

*r),* expected heterozygosis (*H_E_*), and population differentiation (*F_ST_*) are shown. Location codes are presented in Table 1. Values of average allele richness (

**Figure 2:**
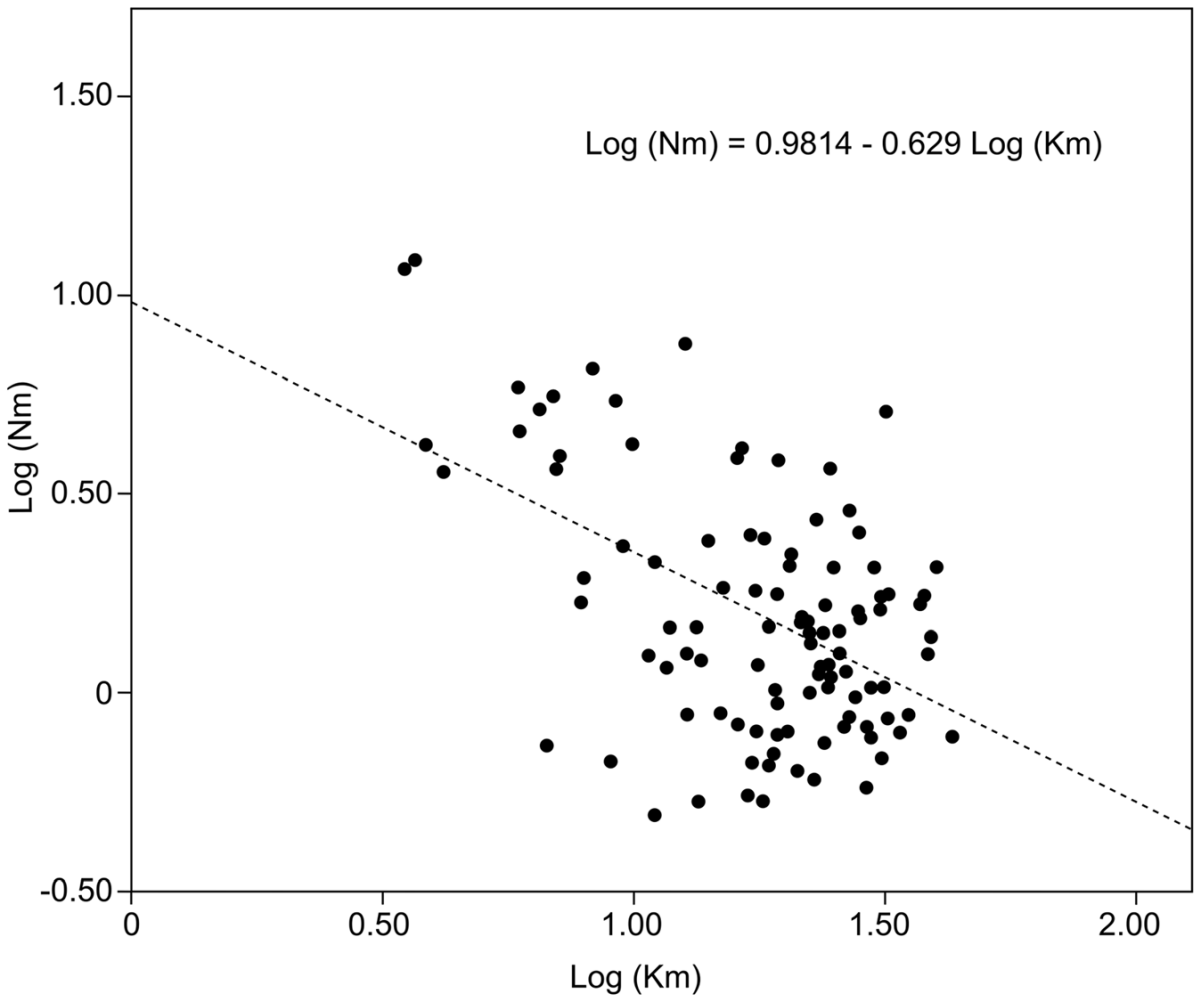
Linear regression of estimates of the effective number of migrants (*Nm*) and geographical distances between population pairs (both variables log-transformed, see Methods).

Genetic and geographical distance matrices were positively correlated across all three watersheds (*r* = 0.326, *P*<0.001). Within watersheds, only the Fluvià River displayed a significant correlation (Fluvià River, *r* = 0.694, *P*<0.01). Significant negative regression of the log-transformed effective number of migrants and geographical distances were detected for the whole data set of the studied locations in Spain. Furthermore, as the slope of the regression (b = –0.629) was closer to –0.5 than to –1, a two-dimensional stepping stone model better explained the population relationships (Fig. 2). Within watersheds, regression analyses of the number of migrants and distances were only significant for the Fluvià River, where a slope of –1.101 supported a one-dimensional stepping stone model. Marginal significance (*P* = 0.052) was obtained for the whole Ter River basin (b = –0.455); however, this significance disappeared when the analysis focused on the mainstem of the river (CL, VE, TO, and TV locations).

**Figure 3:**
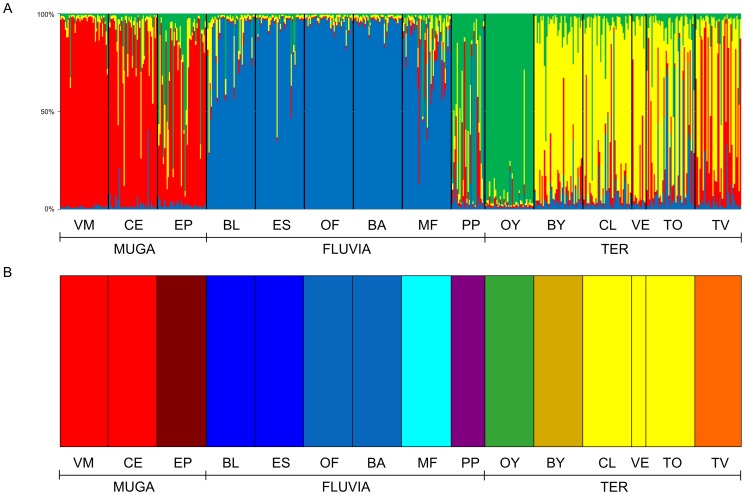
Bayesian analyses of population structure. Analyses were carried out with (*a*) STRUCTURE and (*b*) BAPS in the Iberian *G. holbrooki* populations. In (*a*) each individual is represented as a vertical bar partitioned into segments of different color according to the proportion of the genome belonging to each of the four identified clusters (*K* = *4*). In (*b*) each location shows a different color according to the cluster to which it belongs. Location codes are presented in Table 1.

**Figure 4:**
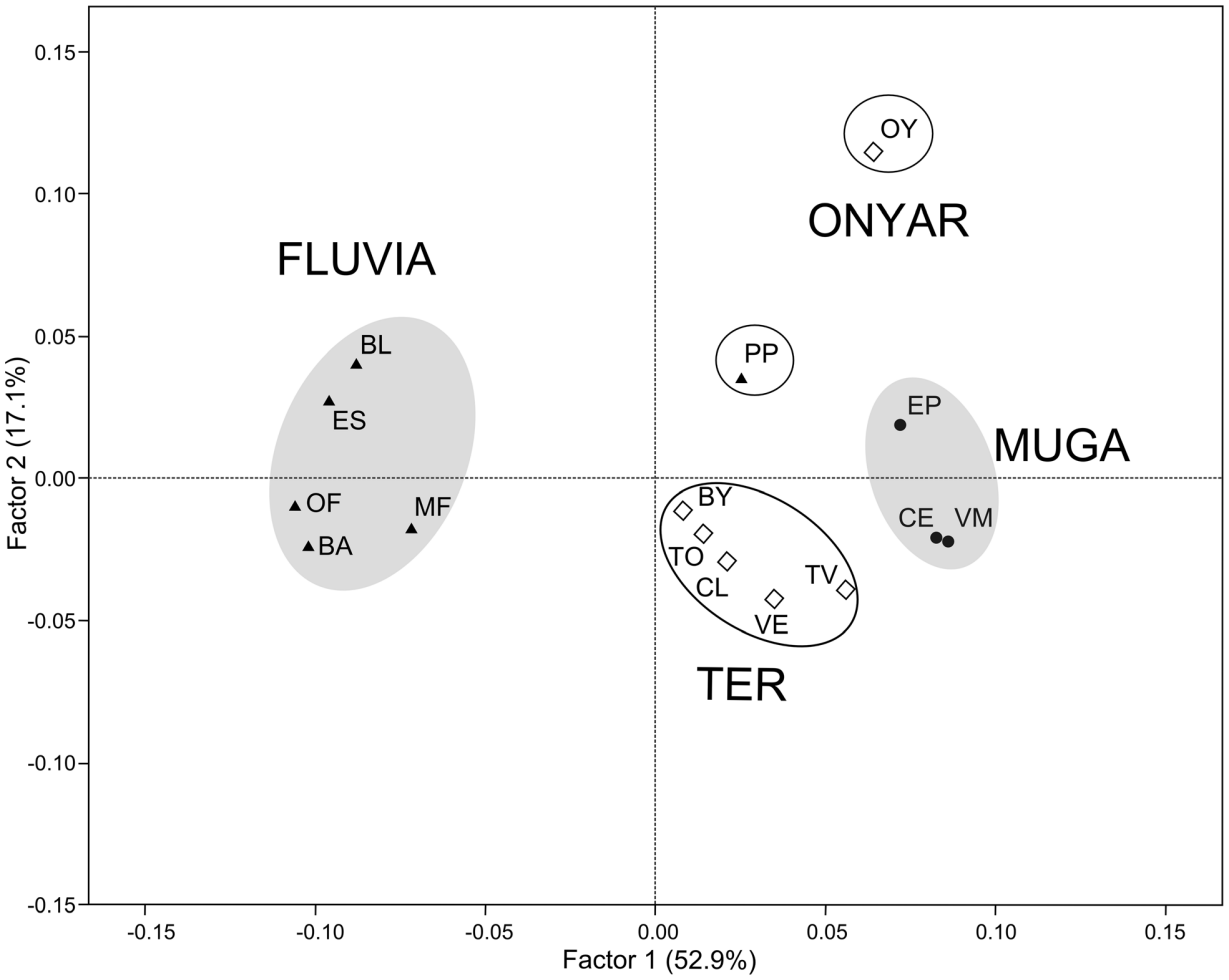
Principal component analysis (PCA) showing the relationships among the studied *G. holbrooki* populations. Samples are projected onto the plane formed by the first two principal axes. The first factor explained the 52.9% of total variance, the second 17.1%, and the third 13.5%. Empty circles indicate positive values of the third axis, while grey circles indicate negative values. Location codes are presented in Table 1.

Although the number of clusters identified varied among analyses, the clusters basically reflected population relationships according to the extent of drainage connection. Evanno’s method indicated four STRUCTURE clusters (Fig. 3a). Cluster 1 grouped individuals from all sites in the Muga river basin, while cluster 2 grouped all samples from the Fluvià River watershed, except for PP. The third cluster grouped all samples from the Ter River populations, except the Onyar tributary, which was assigned to cluster 4. The coastal locations of EP, PP, and TV showed a remarkable degree of cluster admixture. The Monmonier’s algorithm of BARRIER identified four barriers supported by at least six loci (Fig. 1). The first (*a*) and third (*c*) barriers reflected the isolation of the three watersheds, with the exception of the PP location in the Fluvià River basin, which was grouped with the Muga River collections. The second (*b*) and fourth (*d*) barriers reflected the distinct genetic composition of the OY and BY locations separated from the mainstem of the Ter River by river transects where mosquitofish were not detected during our surveys. Overall, these results mainly agreed with the population relationships depicted by the two principal axes of the PCA analysis (Fig. 4). The first axis explained 52.9% of the allelic variance, and clearly differentiated the Fluvià River collections from the rest, with the exception of PP. The second axis (17.1%) separated the Muga and Ter River basins from the singular population of OY. BAPS identified 10 homogenous units within the study region, basically indicating that each collection represented a single panmictic group (Fig. 3b). Only intra-basin locations that had the largest estimates of current gene flow (Table 5) were grouped together; specifically, VM and CE in the Muga basin, BL and ES and OF and BA in the Fluvià basin, and CL, VE, and TO in the Ter basin.

**Table 5:** BAYESASS estimated migration rates among locations.

	From:
To:	VM	CE	EP	BL	ES	OF	BA	MF	PP	CL	VE	TO	TV	BY	OY
VM	*0.8895*	0.0102	0.0173	0.0057	0.0061	0.0055	0.0057	0.0058	0.0076	0.0067	0.0066	0.0078	0.0088	0.0074	0.0092
CE	**0.1636**	*0.7131*	0.0119	0.0066	0.0079	0.0067	0.0083	0.0097	0.0069	0.0097	0.0069	0.0096	0.0218	0.0086	0.0086
EP	0.0194	0.0182	*0.8618*	0.0061	0.0064	0.0061	0.0059	0.0063	0.0096	0.0077	0.0059	0.0088	0.0212	0.0071	0.0094
BL	0.0061	0.0061	0.0065	*0.8848*	0.0127	0.0109	0.0094	0.0076	0.0073	0.0077	0.0060	0.0073	0.0060	0.0151	0.0065
ES	0.0063	0.0064	0.0065	**0.1882**	*0.6862*	0.0090	**0.0423**	0.0090	0.0062	0.0075	0.0062	0.0070	0.0065	0.0064	0.0062
OF	0.0062	0.0062	0.0060	0.0131	0.0298	*0.6898*	**0.1932**	0.0102	0.0062	0.0068	0.0064	0.0066	0.0061	0.0073	0.0062
BA	0.0061	0.0057	0.0060	0.0216	**0.0611**	0.0210	*0.8234*	0.0100	0.0060	0.0067	0.0054	0.0075	0.0064	0.0069	0.0063
MF	0.0079	0.0077	0.0080	0.0250	**0.1091**	0.0190	**0.0509**	*0.7184*	0.0074	0.0085	0.0061	0.0091	0.0084	0.0074	0.0070
PP	0.0095	0.0110	**0.0634**	0.0112	0.0269	0.0094	0.0114	0.0179	*0.6908*	0.0210	0.0087	0.0288	0.0201	0.0123	**0.0574**
CL	0.0103	0.0201	0.0166	0.0096	0.0136	0.0085	0.0082	0.0116	0.0081	*0.8060*	0.0073	0.0225	0.0214	0.0268	0.0095
VE	0.0175	0.0142	0.0134	0.0136	0.0139	0.0133	0.0132	0.0133	0.0135	**0.1247**	*0.6804*	0.0244	0.0148	0.0173	0.0126
TO	0.0108	0.0084	0.0296	0.0251	0.0289	0.0086	0.0129	0.0093	0.0085	**0.0714**	0.0066	*0.7393*	0.0133	0.0191	0.0084
TV	0.0161	0.0253	0.0171	0.0064	0.0078	0.0061	0.0073	0.0077	0.0078	0.0324	0.0069	0.0177	*0.8260*	0.0081	0.0075
BY	0.0073	0.0068	0.0072	0.0072	0.0069	0.0073	0.0062	0.0061	0.0080	0.0087	0.0063	0.0119	0.0097	*0.8935*	0.0067
OY	0.0062	0.0067	0.0104	0.0062	0.0076	0.0076	0.0068	0.0077	0.0078	0.0073	0.0059	0.0082	0.0070	0.0113	*0.8933*

Diagonal values (in italics): Proportions of non-migrant mosquitofish. The most relevant migration rates are shown in bold (see Results for further explanation).

Hierarchical AMOVAs revealed that the genetic variance was significant at all levels, with most of the variance being attributed to individuals within locations (80.9–82.9%). In the hydrographical model, the variance assigned to divergence among populations within basins (8.8%) was smaller compared to the variance among river basins (10.3%). This pattern reflected the above noted population divergence among and within drainages. In the AMOVA based on the four STRUCTURE clusters, the proportion of genetic variance explained within clusters decreased to 6.8%, while the variance among these clusters increased (12.3%), probably reflecting the distinctiveness of the OY collection from locations in the mainstem of the Ter River.

Overall, contemporary dispersal rates indicated an average of 21.4% of immigrant individuals at each location (range 11–31%, Table 5). As a conservative rule, we only discussed the 5% of highest estimates (11 out of 210 values). Within this framework, the most significant estimates of dispersal rates were basically downstream within rivers. Only at the ES site, and particularly at the OF site, a significant proportion of individuals were upstream immigrants from BA. BAYESASS also indicated the presence of gene flow among river basins, particularly from EP (Muga River) to PP (Fluvià River), and from the Onyar River (OY, Ter basin) to PP (Fluvià River).

## Discussion

### Genetic diversity and invasive potential retained in Spanish basins

In colonized territories, the level of genetic diversity of the invading species is expected to be reduced compared to original sources as a result of founder events [4], [7], [33], [56]. This phenomenon has been suggested for European mosquitofish populations when compared against the American collections from Florida [57]. For a more accurate evaluation of the effect of founder events on the genetic diversity and evolutionary potential of the Spanish populations, we compared the level of genetic diversity in the Spanish populations against those observed in the American populations considered to be the potential sources of the fish that were introduced to Europe. Previous studies have shown that the haplotype Hol1 is almost fixed in the Spanish collections and the American populations of Brunswick and Potomac River [16]. In addition, the Potomac River collection was the most closely related to European mosquitofish in a survey based on six microsatellites [58]. Genetic diversity declines by a factor of (1-^1^/_2_ *Ne*) per generation during a founder effect depending on the effective number (*Ne*) of introduced individuals (see for instance [4]). Historical records indicate that just 12 individuals of mosquitofish were introduced to Spain [59]. If we consider the best case scenario for diversity retention involving just a single generation founder effect with an effective population size of 12 individuals, the population should have preserved around 95% of the original genetic diversity, or even more if some of the specimens were gravid females, because multiple paternity increases the effective size in mosquitofish populations [23]. With no relevant effects of mutations on population structure (*F_ST_* = *R_ST_*), all of the diversity present in the invaded range should be attributed to the population sources of the invasion. Based on the average diversity in the two American collections studied here (*H_E_* = 0.522, Table 4), the estimated diversity at these source locations agreed with the estimated total diversity at the Spanish study region (*H_T_* = 0.522). This observation indicates minimal, if any, loss of genetic diversity in the introduced populations of the Iberian Peninsula. Nevertheless, a significant reduction in allele richness was detected at the invaded Spanish locations, because this parameter is more sensitive to bottlenecks compared to average heterozygosity [24], [60]. It is therefore likely that introduced Spanish populations have not substantially reduced their evolutionary potential compared to American sources, because the levels of additive variance might still be less sensitive to bottlenecks compared to neutral variation (see reviews in [36], [61]). For example, introduced Australian populations of the guppy (*Poecilia reticulata*) showed strong genetic bottlenecks in genetic diversity when measured with neutral markers; yet, these populations retained substantial additive variation [62].

Lower neutral genetic variation is often detected in populations at the limit of the distribution range [63]. Reduced diversity and singular mtDNA haplotypes of *G. holbrooki* in northern American drainages indicated postglacial colonization from refuge populations in Georgia or Florida [64]. A recent work based on microsatellite variation confirmed an important reduction in allele richness (up to 50%) and heterozygosity (up to 30%) of American *G. holbrooki* populations in North Carolina and northward compared to populations that occurred to the south in South Carolina and Florida [58]. Nevertheless, peripheral populations often display greater stress-adaptation favoring subsistence in unstable environments [65]. Hence, available information about species with broad distributions indicates that less-stable habitats within native ranges serve as frequent sources of invasive populations (see [66]). If this was the case for mosquitofish, marginal populations of the northward range of America might have already acquired the evolutionary changes to be invasive during the postglacial period, as far as substantial additive variation could be retained during related founder effects despite losses of neutral genetic variation. Therefore, the American mosquitofish sources used in the European introduction might represent an “invasive bridgehead”. As defined by Lombaert et al. [67], invasive bridgeheads are particularly successful invasive populations that serve as the source of colonists for remote new territories. Because genetic diversity in the Spanish populations was not significantly reduced during the introduction, enough additive variance to respond to novel selection pressures in these non-native environments was probably conserved, favoring the successful and quick expansion of the species throughout the entire Mediterranean basin documented in historical records [20].

### Dispersal patterns and population diversity in invaded locations

Precise historical records are not available about the introduction of mosquitofish to the study basins. While *G. holbrooki* was first introduced to southwestern Spain in 1921, it was absent from the study basins in 1942 when insecticides (DDT) replaced mosquitofish as the major agent against malaria vectors. Malaria was eradicated in 1964 from Spain; however, mosquito control, including mosquitofish introductions, continued (reviewed in [68]). Mosquitofish were apparently introduced to Lake Banyoles between 1952 and 1964, after they had already become established in other parts of the study watersheds [69]. Given that mosquitoes are abundant in marshlands dominating the lowlands of the three river basins [70], mosquitofish were probably first introduced into these lowland areas. The mosquitofish in the study river basins probably originated from well-established populations in central and southern Catalonia, such as the deltas of Llobregat and Ebro rivers, where mosquitofish were already present by 1942 [68]. In the Ebro River, which is located around 300 km south of our study area, mosquitofish populations exhibit similar levels of total diversity (*H_T_* = 0.532), with this diversity mainly being distributed within locations (*H_E_* = 0.523)(Diez-del-Molino et al. unpublished).

Significant genetic divergence among study locations indicated the isolation of current mosquitofish populations both within and among the three studied basins. According to Smith et al. [29], American mosquitofish populations along a river basin displayed a pattern of population divergence resulting from genetic drift and gene flow. However, some complex microgeographic patterns were also present as a result of interactions between dramatic demographic fluctuations and breeding structures complicated by multiple insemination and differential sex and cohort dispersal ability [71]. Larger divergence among mosquitofish populations located in the upper reaches of the study basins might be related to founder events during dispersal along the basins, because the contribution of mutations to population structure was not significant. The average level of population diversity at these locations represented 85% of the total genetic diversity in the area. At each location, the stated percentage indicated 4–5 generations of bottlenecks from just 12 individuals in magnitude (1-^1^/_2_ *Ne* per generation of diversity losses), or more bottleneck-generations with larger *Ne*. In fact, signals of recent bottlenecks were detected at 30% of the locations, and affected populations from all river basins.

In the lowlands, differentiation among *G. holbrooki* populations exhibited similar patterns to that observed for the endemic killifish *Aphanius iberus*, in which increased gene flow was observed between populations during floods [72], [73]. If flooding also connects mosquitofish populations, this process alone justified why barriers were not detected between the downstream mosquitofish populations in the Muga and Fluvià rivers, because the mouths of both rivers flow out of the same marshland (Aiguamolls de l’Empordà). In addition, substantial reductions in the population size of mosquitofish have been reported to recover within a few months after flooding [74]. Moreover, pregnant females might buffer associated genetic bottlenecks [59], [75]. In the basins studies here, larger population divergence (*F_ST_* = 0.46) has been detected among remaining native populations of the three-spine stickleback (*Gasterosteus aculeatus*) [76]. While stickleback remains in unpolluted streams with abundant aquatic vegetation [77], mosquitofish are successful invaders of modified and disturbed habitats, such as ponds, irrigation ditches, and modified stream channels in urban areas [15]; such habitats allow increased gene flow among locations in invaded basins (e.g., Diez-del-Molino et al. unpublished).

BAYESASS indicated current relevant migration rates between some neighboring population pairs in all of the study basins. This phenomenon resulted in BAPS clustering the VM and CE locations in the Muga River, the BL and ES and the BA and OF in the Fluvià River basin, and the CL and VE in the Ter river basin. These location pairs were separated by a distance of 5.3 km on average (range 3.50–8.28), with significant dispersal occurring both downstream and upstream (BA to OF). High positive spatial autocorrelation of allele frequencies at hydrological distances of 6–150 km has been observed in American drainages (reviewed in [29]), indicating gene flow between distant locations within short time scales (few generations). In [78], the authors suggested that mosquitofish may disperse at rates greater than 800 m/day in unimpeded corridors. These observations indicate that the dispersal ability of mosquitofish is sufficient to colonize an entire basin from a single founder effect. Once a single population is established, further active upstream or passive downstream dispersal leads to the founding of new populations, and maintains high enough gene flow to preserve existing genetic diversity throughout all locations along the invaded river basin, and to overcome founder effects.

In the Spanish basins, isolation by distance was indicated among mosquitofish populations through the significant correlation between genetic and geographical distances in the whole territory. The significant negative relationship (b = –0.629) observed between the log-transformed effective number of migrants (Nm) and the geographical distances also supported a two-dimensional stepping stone model of gene flow. Thus, in addition to active linear and flood-mediated dispersal along river basins, human-mediated translocations between road-neighboring populations from separate basins have probably contributed to the spread of mosquitofish in the area (for instance to OY and BY in the Ter river basin). In Australia, unreported and unregulated human-mediated dispersal has led to the introduction of mosquitofish to areas outside of its first sites of introduction [26]. In addition, a higher incidence of aquarists in more densely human populated areas (such as Girona in this study) might contribute to mosquitofish dispersal, through aquaria fish being discarded into urban ponds and river streams. Such practices have contributed to the dispersal of alien poeciliid species in Australia [79] and Spain [18]. Human-mediated translocations of endangered native species, such as *A. iberus* and *G. aculeatus*, are forbidden by Spanish laws directed towards protecting biodiversity. However, human-mediated dispersal might represent a major means of promoting gene flow between distant populations of mosquitofish, with such dispersal probably contributing towards retaining the high levels of genetic diversity within the populations of this species throughout the whole territory. It is also likely that human-mediated dispersal plays an important role in the maintenance of the invasive potential of these introduced populations, enabling them to outcompete the native fish.
